# Identification of Hidden Key Hepatitis C Populations: An Evaluation of Screening Practices Using Mixed Epidemiological Methods

**DOI:** 10.1371/journal.pone.0051194

**Published:** 2012-12-07

**Authors:** Angelique P. A. Vermeiren, Nicole H. T. M. Dukers-Muijrers, Inge H. M. van Loo, Frans Stals, Dirk W. van Dam, Ton Ambergen, Christian J. P. A. Hoebe

**Affiliations:** 1 Department of Sexual Health, Infectious Diseases and Environmental Health, South Limburg Public Health Service (GGD Zuid Limburg), Geleen, The Netherlands; 2 Department of Medical Microbiology, School of Public Health and Primary Care (CAPHRI), Maastricht University Medical Center (MUMC+), Maastricht, The Netherlands; 3 Department of Medical Microbiology, Atrium Medical Center Parkstad Heerlen, Heerlen, The Netherlands; 4 Department of Medical Microbiology and Infection Control, Orbis Medical Center, Sittard, The Netherlands; 5 Department of Methodology and Statistics, Faculty of Health, Medicine and Life Sciences, Maastricht University, Maastricht, The Netherlands; Duke University, United States of America

## Abstract

**Background:**

Hepatitis C virus (HCV) is a major cause of liver diseases worldwide. Due to its asymptomatic nature, screening is necessary for identification. Because screening of the total population is not cost effective, it is important to identify which risk factors for positivity characterize the key populations in which targeting of screening yields the highest numbers of HCV positives, and assess which of these key populations have remained hidden to current care.

**Methods:**

Laboratory registry data (2002–2008) were retrieved for all HCV tests (23,800) in the south of the Netherlands (adult population 500,000). Screening trends were tested using Poisson regression and chi-square tests. Risk factors for HCV positivity were assessed using a logistic regression. The hidden HCV-positive population was estimated by a capture-recapture approach.

**Results:**

The number of tests increased over time (2,388 to 4,149, *p*<.01). Nevertheless, the positivity rate among those screened decreased between 2002 and 2008 (6.3% to 2.1%, *p*<.01). The population prevalence was estimated to be 0.49% (95%CI 0.41–0.59). Of all HCV-positive patients, 66% were hidden to current screening practices. Risk factors associated with positivity were low socio-economic status, male sex, and age between 36–55. In future screening 48% (95%CI 37–63) of total patients and 47% (95%CI 32–70) of hidden patients can be identified by targeting 9% (men with low socio-economic status, between 36–55 years old) of the total population.

**Conclusions:**

Although the current HCV screening policy increasingly addresses high-risk populations, it only reaches one third of positive patients. This study shows that combining easily identifiable demographic risk factors can be used to identify key populations as a likely target for effective HCV screening. We recommend strengthening screening among middle-aged man, living in low socio-economic neighborhoods.

## Introduction

Hepatitis C virus (HCV) is a major cause of chronic liver diseases worldwide [1]. In industrialized countries, HCV is the leading cause of liver transplantation [1]. Testing is necessary for identifying infected persons because both acute and chronic infections are asymptomatic in up to 70% of cases [2], [3]. Because treatment for HCV has improved greatly, with cure rates up to 90% for genotypes 2 and 3 [2], [4], the identification of HCV-infected individuals has become even more imperative.

The Centers for Disease Control and Prevention (CDC) recommends that persons meeting the following criteria be screened for HCV: if they ever injected illegal drugs; were ever on long-term dialysis; were born to an HCV-positive mother; are healthcare, emergency medical or public safety workers who have been in contact with HCV-positive blood; received a blood transfusion or organ transplant before July 1992 or clotting factor concentrates produced before 1987 [3]. However, only focusing on people with a history of possible exposure to HCV-infected blood or needles is not sufficient because it has been found that 9% to 21% [5]–[7] of HCV-infected people do not report a history of blood/needle contact. Modeling studies in the USA have further estimated that many HCV-positive individuals remain untested under current screening practices [8], [9], with estimates up to 78% [10]. Therefore, the CDC advises determining the proportion of unscreened positives (hidden population), indicating missed opportunities for the identification of persons with selected risk factors for HCV infection [5].

The standard screening policy in the Netherlands is in line with CDC standards and focuses on high-risk groups and people showing HCV-related symptoms [11]. Prevalence estimates in the Netherlands range between 0.1% and 0.4% and are based on extrapolation of the prevalence in high- and low-risk groups, except for three smaller studies that used random population sampling and sampling of GP patients [12]–[15]. Performing a capture-recapture analysis to improve estimates has been advised [16]. Since 2007, some (mostly regional) screening interventions have been launched to improve case findings [17], [18]. The (economic) evaluation of these programs found that only active screening in groups with high HCV positivity was cost effective, which is comparable to findings in the UK and the USA [9], [19]–[21].

Risk factor studies on screening in England [22], France [23], and the USA [10] have indicated risk factors for HCV positivity corresponding to CDC recommendations and added low socio-economic status (SES) and age between 35–55 years. The CDC currently considers the screening of a 1945 through 1965 birth cohort [24], [25] These demographic risk factors (as compared to ‘history of needle/blood contact’), have the advantage of being more practically applicable in screening programs. Nevertheless, previous studies have not identified which combination of factors characterize the key populations in which screening would yield the highest numbers of HCV positives. Furthermore, it is important to assess whether these key populations are hidden to current care to facilitate the efficient screening and treatment of HCV.

By using mixed epidemiological methods including a capture-recapture approach on surveillance data (2002–2008) of three laboratories performing all of the HCV tests in the southern part of the Netherlands (population 0.5 million), we aim to inform a more effective screening. Therefore we assess 1. time trends in number of tests and positivity rate; 2. factors associated with HCV testing and positive results, and 3, we aim to estimate the number of hidden HCV cases and combine risk factors to identify key populations in which screening would yield to the most (hidden) HCV cases to be found.

## Methods

### Ethical Approval

The medical ethics committee of the Maastricht University Medical Centre (Maastricht, the Netherlands) approved the study (11-4-136) and waived the need for consent to be collected from participants. Since retrospective data originated from standard care (in which one can opt-out for the use of their data for scientific research) and were analyzed anonymously, no further informed consent for data analysis was obtained.

### Study Population

We performed a retrospective cohort study based on all of the laboratory tests between January 1^st^, 2002, and December 31^st^, 2008, provided by the three hospital laboratories that perform the HCV tests in the region of South Limburg, the Netherlands. The study region had 520,552 adult (≥18 years) inhabitants in 2002 and 500,955 adult inhabitants in 2008 [26].

The available data included age (categories: 18–25, 26–35, 36–45, 46–55, 56–65, and 65+ years old), sex, postal code, test date, test result, and care provider. The subjects were screened by their general practitioner (GP), specialist, or non-hospital, non-GP care providers, which included the following: nursing homes (approximately 40%), addiction health services (approximately 30%), public health services (approximately 20%), and occupational health services (approximately 10%). As indicators of SES, the percentage of people between 15 and 65 years old receiving social welfare (SESsw) in the neighborhood (retrieved from Statistics Netherlands [27], comparable to census tract data) and the mean property value within the neighborhood (SESpv) were matched to the postal codes of the tested subjects. Both of the SES indicators (correlation ρ = 0.59) were split into tertiles at the neighborhood level (low SESsw: ≥28%; middle: 23%–28%; high: ≤23%; SESpv low: ≤174 k Euro; middle: 174 k–215 k Euro; high: ≥215 k Euro). Tests without a recorded valid postal code performed by institutes for the homeless, addiction health services, or in prisons were attributed to the lowest SES levels (n = 160, 34 positives).

We matched the data from the three laboratories by sex, date of birth, and postal code. We coded the resulting data (28,827 tests). We removed nine tests because of missing sex or birth dates, and 3,415 tests because subjects resided outside the study area. Furthermore, we removed 561 tests because of missing and/or invalid postal codes. In total, 24,842 tests were used, of which 1,865 (823 unique patients) were positive.

### Diagnostic Criteria

HCV antibodies were detected with an ELISA (AXSYM version 3.0, Abbott, Chicago, USA) according to screening procedures in the Netherlands. Confirmation was performed with a recombinant immunoblot assay (Deciscan HCV plus, Bio-Rad, Hercules, CF, USA) and/or polymerase chain reaction (PCR, COBAS Amplicor, Roche, Branchburg, NJ, USA). When an acute infection is suspected or when the patient is HIV positive or on hemodialysis, only PCR is used for screening (104 cases). In the current study, we defined a positive confirmation test or PCR as a positive case. We thus considered four ELISA-positive tests that were not confirmed, as negative.

### Statistical Analyses

#### Current screening trends

To assess screening trends, we defined screening tests as all negative tests for a subject and the first positive test (23,800 tests included in the analysis). We used Chi-square linear-by-linear tests to examine trends in the positivity rate among the tested population and the proportion of the total population that received testing.

To evaluate which factors were associated with testing, a generalized linear model with Poisson distribution was applied to the screening test data, in which the variables sex, age category, test year, care provider, SESsw and SESpv were entered by a forward procedure.

#### Factors associated with HCV testing and positivity

To identify risk factors for HCV positivity, we applied logistic regression modeling using all of the screening tests with the same variables that were used in the Poisson model. We examined all two-way interactions with care provider and test year. Statistically significant interactions are only presented when opposite trends, tested by Poisson distribution, were noted in subgroups or when trends were only apparent in a particular subgroup.

To evaluate whether the results were biased by an unequal distribution of drug users among the subgroups, analyses were repeated without the tests performed by addiction health services (a separate ‘drug use’ variable could not be made because specific information on HCV-negative tests at non-hospital, non-GP test sites was only available between 2002–2006).

#### Hidden key populations

To calculate the number of hidden HCV-positive individuals, data from the 1,865 positive tests (823 patients) were used in a capture-recapture model [28], [29]. Within this analysis, the overlap in patients between different care provider categories (GPs, specialists, and non-GP, non-hospital) was used to estimate the number of HCV positives not identified by any of the three care providers (hidden population). First, we estimated the most parsimonious model by applying a generalized linear model with Poisson distribution (accounting for the different possibilities of interactions between the care providers in the model). Second, we estimated the number of non-tested individuals using the most parsimonious model. Assumptions of the capture-recapture method are considered in the discussion section. We based subpopulations on sex, age, SESpv and SESsw. The prevalences in each subgroup were calculated by dividing the estimated number of HCV positives in the population by the total population in each subgroup, based on data from Statistics Netherlands, 2007 [26], [27].

We repeated the capture-recapture analysis without any of the tests performed by addiction health services.

Lastly, based on risk factors for positivity found, we calculated by capture-recapture which screening scenarios would yield most (hidden) HCV positives and the number needed to screen in each screening scenario to detect one case of HCV.

We used SPSS 17.0 for the analyses. Significance levels lower than *p* = 0.05 were considered to be statistically significant.

## Results

From 2002 to 2008, 23,800 screening tests were performed among 17,137 persons, of whom 823 (3.5%) were HCV positive. Over half of the screening tests were performed among men (54.9%), and the median age was 48 years (interquartile range 35–63 years).

### Current Screening Trends

**Figure 1:**
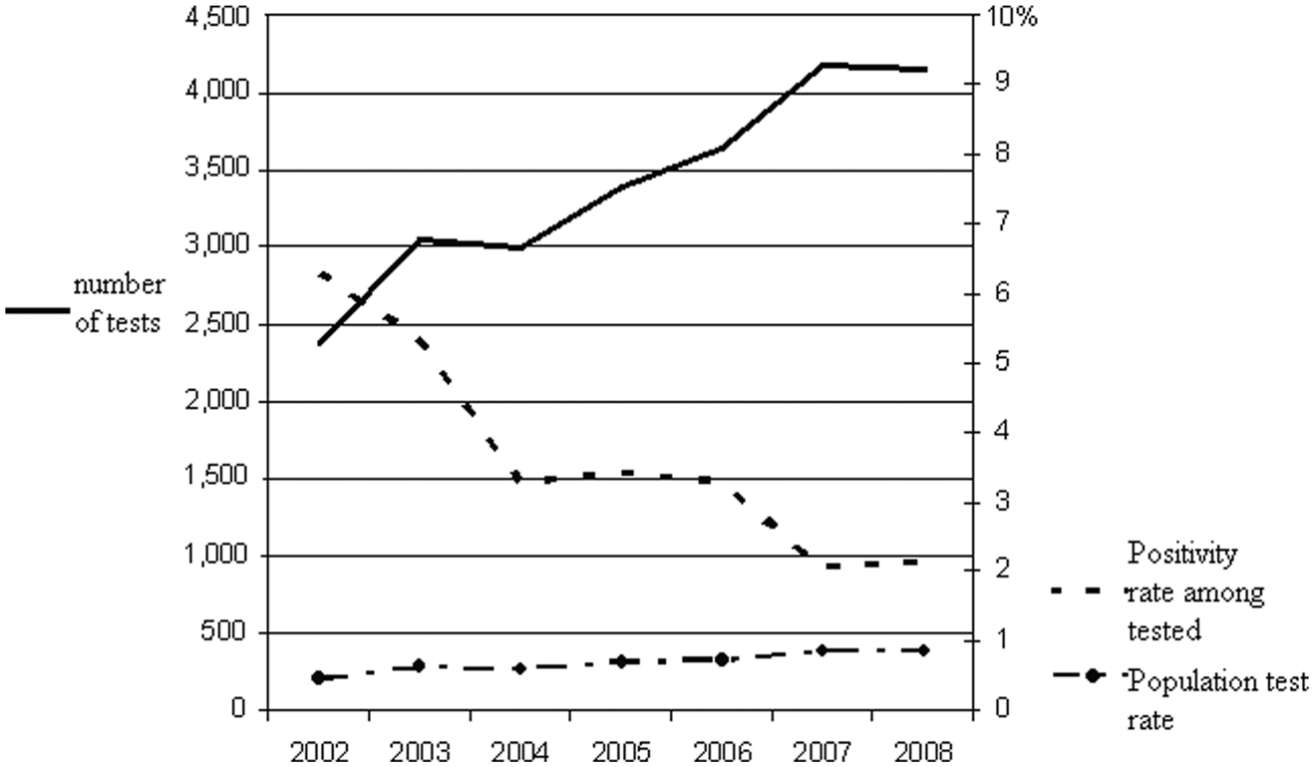
The total number of HCV tests, the positivity rate and the population test rate. Laboratory surveillance data, South Limburg, the Netherlands, 2002–2008.

Over time, the number of HCV screening tests increased from 2,388 to 4,149 (*p*<.01), which translated to the screening rate among the total population per year increasing from 0.46% to 0.83% (*p*<.01) (Figure 1). By contrast, the positivity rate among those screened showed a negative trend (6.3% to 2.1%, *p*<.01). Although the number of HCV screening tests increased significantly among all age categories, the negative trend in positivity was only significant in persons less than 45 years old (0.3% to 0.1%, 9.6% to 1.7%, and 14.8% to 7.7% in the 18–25, 26–35, and 36–45 age category, respectively), not among the older groups (6.3% to 5.3%, 2.0% to 0.8%, and 0.2% to 0.6% for the 46–55, 56–65, and 65+ age groups, respectively).

#### Factors associated with HCV testing and positivity

Men were screened more often than women (Table 1). Most tests were performed among people residing in low-SES neighborhoods (both SESpv and SESsw) followed by the middle-SES groups. Most tests by specialists were performed in the oldest (65+) group, whereas GP and non-hospital, non-GP screening providers tested the 26- to 45-year-old group the most. The factors for screening did not change significantly over time.

**Table 1:** Factors of HCV testing (GLM, Poisson) and factors of HCV positivity (logistic regression).

		Screening per subpopulation		Risk factors	
		Number of tests	RR adjusted^a^ (95%CI)	Positives (%)	OR adjusted^a^ (95%CI)
Sex	Male	13,062	1.18 (1.17–1.19)**	573 (4.4)	1.71 (1.46–2.00)**
	Female	10,738	1.00	250 (2.3)	1.00
Age	18–25	2,055	1.00	21 (1.0)	1.00
	26–35	4,182	1.69 (1.63–1.76)**	158 (3.8)	4.08 (2.57–6.47)**
	36–45	4,614	1.84 (1.77–1.90)**	354 (7.7)	8.95 (5.72–13.99)**
	46–55	4,057	1.64 (1.58–1.70)**	217 (5.3)	6.40 (4.06–10.10)**
	56–65	3,606	1.64 (1.58–1.69)*	47 (1.3)	1.75 (1.03–2.95)*
	>65	5,286	2.84 (2.75–2.95)**	26 (0.5)	0.64 (0.36–1.14)
Care provider	GP	4,470	1.00	160 (3.6)	1.00
	Specialist	16,515	2.66 (2.61–2.72)**	390 (2.4)	0.90 (0.74–1.09)
	Other^b^	2,815	0.68 (0.65–0.71)**	273 (9.7)	3.13 (2.54–3.86)**
SES % on	Low SES	13,338	2.24 (2.17–2.31)**	609 (4.6)	1.76 (1.33–2.29)**
social welfare	Middle SES	5,705	1.11 (1.07–1.15)**	124 (2.2)	0.95 (0.70–1.27)
	High SES	4,757	1.00	90 (1.9)	1.00
SES by property	Low SES	12,664	2.53 (2.44–2.63)**	542 (4.3)	1.50 (1.11–2.04)**
value	Middle SES	7,686	1.33 (1.28–1.38)**	218 (2.8)	1.37 (1.01–1.86)*
	High SES	3,450	1.00	63 (1.8)	1.00

Laboratory surveillance data, South Limburg, the Netherlands, 2002–2008.

a Adjusted for sex, age, care provider, test year, SES by percentage of people on social welfare, and SES by mean property value. * *p*<0.05 **p<0.01.

b Non-hospital, non-GP test providers.

GP: general practitioner; HCV: hepatitis C virus; RR: Relative Risk; OR: Odds Ratio; SES: Socio-economic status.

The percentage of positives among the screening tests varied by sex and age and was highest among men and in those aged 36 to 45 years (Table 1). The highest positivity rate was found among people tested by the non-hospital, non-GP care providers. The positivity rates were higher among residents of low-SES neighborhoods compared with residents of higher-SES neighborhoods. Changes in risk factors for positivity over time were not found.

Analyses excluding screening tests performed at addiction health services showed similar results (data not shown).

### Hidden Key Populations

**Figure 2:**
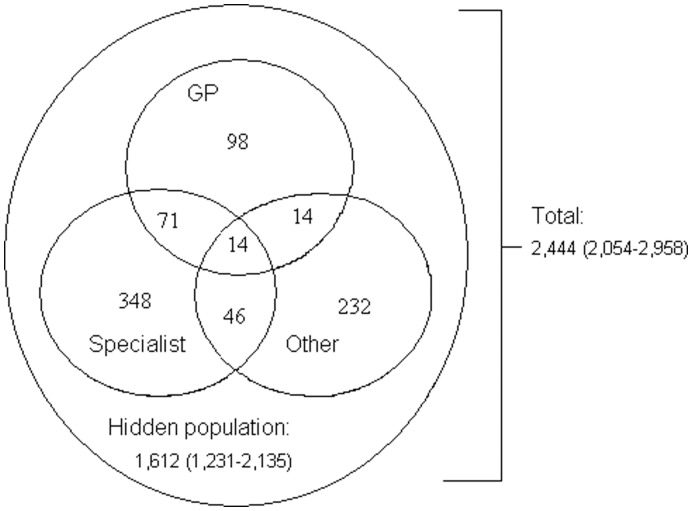
Distribution of HCV cases from the three care providers and the estimated hidden population. Laboratory surveillance data, South Limburg, the Netherlands, 2002–2008.

In the capture-recapture analysis to estimate the size of the hidden HCV-positive population, 1,621 (95%CI 1,231–2,135) HCV positives were not screened and thus hidden for care (Figure 2). As such, 33.7% (95%CI 27.8–40.1) of HCV-positive individuals were screened between 2002 and 2008. Based on this estimation, the prevalence in the total population was 0.49% (95%CI 0.41–0.59). Analysis excluding screens performed at addiction health services produced a prevalence estimate of 0.37% (95%CI 0.30–0.47).

Most hidden HCV positives were found among, men, people aged 36–45 years, residents of low SESsw and low-SESpv neighborhoods, e.g. among men, 1,166 hidden cases lead to a prevalence of 0.71% of total HCV cases among men, and 0.48% prevalence of hidden HCV cases among men (Table 2).

**Table 2:** The number of observed and hidden cases and prevalences in the total and non-screened population.

		Total population^a^	ObservedHCVcases	HiddenHCV cases(95%CI)	Prevalence(95%CI)	Prevalencehidden cases(95%CI)
Sex	Male	244,361	573	1,166 (838–1622)	0.71% (0.58–0.90)	0.48%(0.34–0.66)
	Female	257,922	250	454 (275–748)	0.27% (0.20–0.39)	0.18%(0.11–0.29)
	Sum			1,620 (889–2951)		
Age^b^	18–25	56,158	21	7 (2–24)	0.05% (0.04–0.08)	0.01%(0.00–0.04)
	26–35	65,627	159	269 (152–475)	0.65% (0.47–0.97)	0.41%(0.23–0.72)
	36–45	95,970	352	764 (502–1162)	1.16% (0.89–1.58)	0.80%(0.52–1.21)
	46–55	97,728	218	592 (284–1236)	0.83% (0.51–1.49)	0.61%(0.29–1.26)
	56–65	84,322	47	45 (16–127)	0.11% (0.07–0.21)	0.05%(0.02–0.15)
	>65	102,478	26	4 (2–10)	0.03% (0.03–0.04)	0.00%(0.00–0.01)
	Sum			1,681 (211–13,364)		
SESsw	Low	233,983	609	1073 (784–1468)	0.72% (0.60–0.89)	0.46%(0.34–0.63)
	Middle	141,049	124	333 (156–711)	0.32% (0.20–0.59)	0.24%(0.11–0.50)
	High	127,250	90	170 (85–342)	0.20% (0.14–0.34)	0.13%(0.07–0.27)
	Sum			1,576 (537–4,626)		
SESpv	Low	224,326	542	977 (702–1358)	0.68% (0.55–0.85)	0.44%(0.31–0.61)
	Middle	178,958	218	570 (322–1007)	0.44% (0.30–0.68)	0.32%(0.18–0.56)
	High	98,999	63	25 (9–71)	0.09% (0.07–0.14)	0.03%(0.01–0.07)
	Sum			1,596 (561–4,541)		
Total		502,283	823	1,621(1231–2135)	0.49%(0.41–0.59)	0.32%(0.25–0.43)

Laboratory surveillance data, South Limburg, the Netherlands, 2002–2008.

a Based on population statistics from 2007 [27].

b Age at first positive test.

HCV: hepatitis C virus.

SESsw: Socio-economic status based on % of people receiving social welfare.

SESpv: Socio-economic status based on property value.

In a screening scenario in which all men living in low SESsw between 36 and 55 years would be screened, which is 8.7% of the population, 47.6% (95%CI 37.4–62.8) of the total HCV positives and 47% of hidden cases would be found. In this group, 38 tests would be needed to detect one positive HCV case (Table 3). In a screening scenario in which men living in low SESsw between 36 and 45 year old would be targeted, 31 tests would be needed to detect one positive case (compared to 206 when the total population would be screened).

**Table 3:** Screening scenarios: The percentage of the total population screened, which percentage of the (hidden) total HCV patients will be detected, and the number of people needed to screen.

		% Total^a^ population	% Total cases	% Total hidden cases	Number to screen
			(95%CI)	(95%CI)	(95%CI)
	Total	100	100	100	206 (170–245)
Sex	Men	48.7	71.1 (57.7–89.8)	72.0 (51.7–100)	141(111–173)
	Women	51.3	28.8(21.5–40.8)	28.0 (17.0–46.1)	366(258–491)
Age b	18–25	11.2	1.1(0.9–1.8)	0.4(0.1–1.5)	2006(1248–2442)
	26–35	13.1	17.5(12.7–25.9)	16.6(9.4–29.3)	153(104–211)
	36–45	19.1	45.7(34.9–61.9)	47.1(31.0–71.7)	86(63–112)
	46–55	19.5	33.1(20.5–59.5)	36.5(17.5–76.2)	121(67–195)
	56–65	16.8	3.8(2.6–7.1)	2.8(1.0–7.8)	917(485–1338)
	65+	20.4	1.2 (1.1–1.5)	0.2 (0.1–0.6)	3416 (2847–3660)
SESsw	Low	46.6	68.9(57.0–85.0)	66.2 (48.4–90.1)	139(113–168)
	Mid	28.1	18.7(11.5–34.2)	20.5 (9.6–43.9)	309(169–504)
	High	25.3	10.6(7.2–17.7)	10.5(5.2–21.1)	489(295–727)
SESpv	Low	44.7	62.2(50.9–77.7)	60.3 (54.3–83.8)	148(118–180)
	Mid	35.6	32.2(22.1–50.1)	35.2(19.9–62.1)	227(146–331)
	High	19.7	3.6 (2.9–5.5)	1.5(0.6–4.4)	1145(739–1375)
Combination	Men 36–45	9.5	32.4 (23.5–47.2)	33.8 (20.3–56.1)	59(40–81)
	Men 36–55	19.3	56.5 (44.9–73.3)	56.7 (39.1–82.0)	68(53–86)
	Low SESsw 36–45	8.9	33.2 (24.7–47.0)	33.7 (20.9–54.5)	55(39–74)
	Low SESsw 36–55	18.0	57.3(46.0–73.5)	56.6 (39.5–81.0)	64(50–80)
	Low SESsw men	22.7	48.7(38.7–63.2)	47.5 (32.5–69.3)	96(74–120)
	Men low SESsw 36–45	4.3	19.5(15.1–26.6)	18.1 (11.4–28.7)	31 (25–37)
	Men low SESsw 36–55	8.7	47.6(37.4–62.8)	47.0 (31.6–70.0)	38 (29–48)

Laboratory surveillance data, South Limburg, the Netherlands, 2002–2008.

a Based on population statistics from 2007 [27].

b Age at first positive test.

SESsw: Socio-economic status based on % of people receiving social welfare.

SESpv: Socio-economic status based on property value.

## Discussion

This study applied mixed epidemiological methods to assess (hidden) key populations for HCV screening, using all of the tests performed in a study area. Between 2002 and 2008, the number of screening tests and the screening rate among the population of South Limburg increased (0.47% to 0.84% per year). Simultaneously, the positivity rate among those tested decreased (6.3% to 2.1%). The overall HCV prevalence in the adult population was estimated to be 0.49%; however, 66.3% of all HCV-positive individuals were hidden to current screening. Most tests were performed by specialists, but the positivity rate was the highest in non-GP, non-hospital care settings. Specialists more often screened older people (65+), suggesting opportunistic screening of hospital patients.

Men, persons between 36 and 45 years of age and residents of low-SES neighborhoods had the highest risk of a positive HCV test when screened. Men and residents of low-SES neighborhoods are also the most addressed populations of the current screening policy. Nevertheless, most hidden HCV positives remained to be found among middle age male residents of low-SES neighborhoods. When these three risk factors were combined it was found that targeting only a small fraction of the population (8.7%), would yield the detection of almost half (47%) of hidden HCV cases. Other combinations of risk factors should also be considered, depending on means available. When we applied the birth cohort (1945–1965) screening that is currently under consideration by the CDC, we found that by screening 41% of the population, 54% of the HCV positive populations could be identified, which is less than the 81% in the USA [24], [25].

Although the screening rate increased between 2002 and 2008, the positivity rates decreased. The increase in screening may partly be explained by increased attention concerning HCV, including a campaign promoting HCV testing among risk groups identified by a web-based risk score, which began in April 2007 [17], and a higher screening rate among men reporting sex with men in 2008, which was initiated after this population was found to be at risk in an Dutch outbreak study from Amsterdam [30] (these extra tests did not yield extra HCV-positive cases in our study region). The lower positivity rate over time indicates that the higher proportion of screening has disproportionally more often included persons at lower risk.

Residents of low-SES neighborhoods have previously been found to have higher positivity rates when screened [8], [31]. In this study, two different SES measures were independently associated with higher positivity rates, and this association remained present after excluding tests at addiction care centers. Although low-SES neighborhoods were addressed more in screening, most of the hidden cases are still to be found in these neighborhoods and therefore need to be targeted further. One explanation may be that people with poor health and more HCV risk factors may have a higher probability of living in a low-SES environment. However, previous studies have indicated that even after correcting for major risk factors, SES remains a predictor for HCV positivity [8], [31]. Furthermore, earlier studies have indicated that the source of infection remains unknown in 9% to 21% [5]–[7] of HCV-positive individuals. Unreported drug use might partly explain these results. Future research could include why people with a low SES have a higher HCV positivity rate to enable better prevention and care in these disadvantaged groups.

The estimated prevalence (0.49%) is higher than previous estimations in the Netherlands (0.1% to 0.4%) and lower than the prevalence estimated in Amsterdam (0.6%) [12], [14]. The estimate in this study might be more accurate because this study was the first to use capture-recapture analysis on all of the tests performed within a specific region. A greater number of problematic drug users in the study area (0.45% of the population compared with 0.16% in all of the Netherlands) may also partly explain the higher prevalence [32]. Without the tests performed at addiction health services, the prevalence was estimated to be 0.37% (0.30%–0.47%). However, the number of non-western immigrants in the region, who have a higher HCV positivity rate, [33] is much lower (4.8%) than in the total population of the Netherlands (19.8%) [27].

We acknowledge several limitations of this study. First, we excluded people without a valid postal code in the analysis (except those tested by homeless institutes, addiction health services, or in prison). The positivity rate in this excluded group was higher than in the included group (6.6% versus 3.5%), which may have excluded a vulnerable group and may have led to an underestimation of the prevalence. Nevertheless, the effect is most likely small because we only removed 36 positive persons. Second, distinguishing between a cleared infection and an active infection was not possible because only a small number of people were tested with PCR. Of the 279 subjects who were PCR tested, 231 (82.8%) had an active (acute or chronic) infection, which corresponds to the expected 15%–25% clearing rate [5]. Third, only a limited number of variables could be controlled for due to the limited information available in the data recorded in the laboratories. Last, we recognize that effective screening alone is not sufficient to diminish the HCV disease burden because only 42.8% of the screened HCV-positive individuals consulted a specialist. The number of treated persons will even be lower because not every specialist is certified to provide HCV treatment and not all patients are eligible for treatment. Earlier studies have indicated that it is important to increase awareness among GPs concerning HCV treatment and referral possibilities [18], [34], [35].

For valid results of three-source log-linear capture-recapture models, several assumptions must be met [28], [36]. First, classification and matching have to be correct. Matching was performed by sex, birth date, and postal code. Because information from all of the laboratories covered all three register categories (GP, hospital and non-GP, non-hospital), major misclassification is not expected. The second assumption requiring a closed population could not be fully met because the population was followed for seven years. However, immigration and emigration are low in the study area (<5%) [27]. Furthermore, limiting follow up to two years after the first positive test gave a comparable estimate of the prevalence (0.48%; 95%CI 0.38%–0.63%). The third assumption requires a homogenous population, with no subgroups with markedly different probabilities of being observed and re-observed. Except for an interaction between care provider and age (older people were more often screened by a specialist), no interaction between care providers and the other variables was found, indicating that the different subgroups had an equal chance of being screened by a GP, specialist or non-hospital, non-GP screening facilities. Furthermore, after totaling the estimations of the HCV subgroups in the key population analysis, the non-HCV-tested population (between 1,576 and 1,681) remained close to the original estimate (1,624) (Table 2). Although unlikely, it nevertheless remains possible that subgroups not considered in the analysis would have different probabilities of being registered by any of the three care providers [37], [38].

Although the current HCV screening policy aims to address key populations, many cases remain unidentified. This study shows that combining the easily identifiable demographic risk factors can be used to identify key populations in which HCV screening would be more effective, i.e. screening a small number of people would yield a high detection rate. Based on the results of this study, we recommend strengthening screening among middle-aged man living in low socio-economic neighborhoods. However, efficient testing alone will not be sufficient to diminish the burden of disease related to HCV: consultation with a treatment-providing specialist should be encouraged as well.
